# Antimicrobial Peptide-Functionalized Mesoporous Hydrogels

**DOI:** 10.1021/acsbiomaterials.1c00029

**Published:** 2021-03-15

**Authors:** Saba Atefyekta, Edvin Blomstrand, Anand K. Rajasekharan, Sara Svensson, Margarita Trobos, Jaan Hong, Thomas J. Webster, Peter Thomsen, Martin Andersson

**Affiliations:** † Department of Chemistry and Chemical Engineering, Chalmers University of Technology, SE-412 96, Gothenburg, Sweden; § Department of Biomaterials, Sahlgrenska Academy at University of Gothenburg, Box 412, SE-405 30 Gothenburg, Sweden; ∥ Department of Immunology, Genetic and Pathology, Uppsala University, Rudbeck Laboratory C5, 75185 Uppsala, Sweden; ⊥ Department of Chemical Engineering, Northeastern University, Boston, Massachusetts 02115, United States; # Center for Antibiotic Resistance Research (CARe), University of Gothenburg, SE-40530 Gothenburg, Sweden

**Keywords:** antimicrobial peptides, infection, prevention, hydrogels

## Abstract

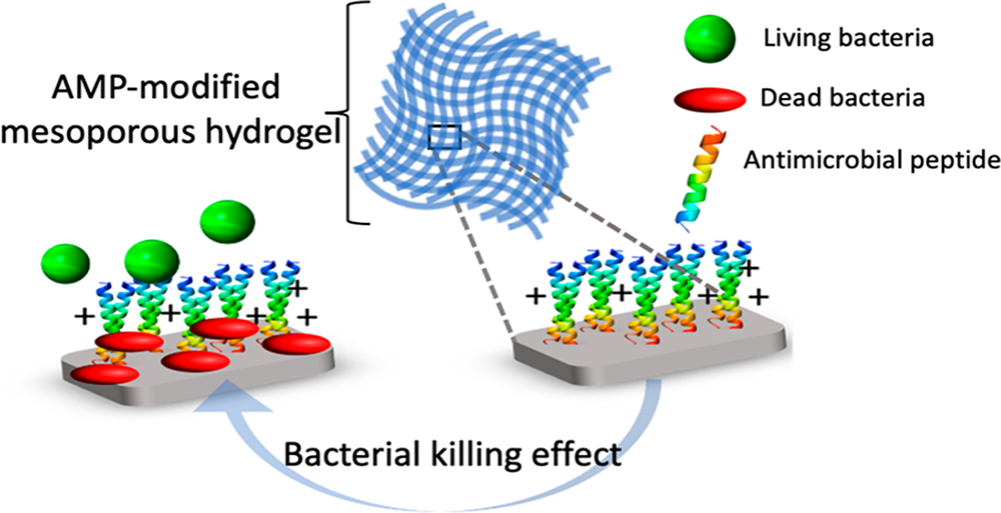

Antimicrobial peptides (AMPs) are seen as a promising replacement to conventional antibiotics for the prevention of skin wound infections. However, due to the short half-life of AMPs in biological environments, such as blood, their use in clinical applications has been limited. The covalent immobilization of AMPs onto suitable substrates is an effective solution to create contact-killing surfaces with increased long-term stability. In this work, an antimicrobial peptide, RRPRPRPRPWWWW-NH2 (RRP9W4N), was covalently attached to amphiphilic and ordered mesoporous Pluronic F127 hydrogels made of cross-linked lyotropic liquid crystals through 1-ethyl-3-(3-(dimethylamino)propyl) carbodiimide (EDC) and *N*-hydroxysuccinimide (NHS) chemistry. The AMP-hydrogels showed high antibacterial activity against *Staphylococcus epidermidis, Staphylococcus aureus*, *Pseudomonas aeruginosa*, methicillin-resistant *S. aureus* (MRSA), and multidrug-resistant **Escherichia coli** for up to 24 h. Furthermore, the AMP-hydrogels did not present any toxicity to human fibroblasts. The AMPs retained their antimicrobial activity up to 48 h in human blood serum, which is a significant increase in stability compared to when used in dissolved state. A pilot *in vivo* rat model showed 10–100× less viable counts of *S. aureus* on AMP-hydrogels compared with control hydrogels during the first 3 days of infection. Studies performed on human whole blood showed that blood coagulated more readily in the presence of AMP-hydrogels as compared to hydrogels without AMPs, indicating potential hemostatic activity. Overall, the results suggest that the combination of amphiphilic hydrogels with covalently bonded AMPs has potential to be used as antibacterial wound dressing material to reduce infections and promote hemostatic activity as an alternative to antibiotics or other antimicrobial agents, whose use should be restricted.

## Introduction

Human skin is an effective barrier protecting underlying tissues from microbial penetration, but it is also the tissue most exposed to damage and injury.^1^ Wounded skin is a vulnerable environment for bacterial colonization and infection.^2^ An infected wound slows down the wound healing process and can result in patient morbidity and high healthcare costs. If an infected wound is not managed properly, it can lead to septicemia and sometimes even death.^3^

Currently, as one of the first steps in the treatment of an infected wound, antibiotics are frequently prescribed to patients despite their ineffectiveness.^4^ However, an effective antibiotic treatment requires microbial identification and careful selection of antibiotics. Microbial analysis is often both a challenging and time-consuming procedure.^5^ In some cases, bacteria present in the wound have the ability to form and exist within a biofilm. Such biofilms have a complex structure and are usually resistant to host immune responses and antibiotic treatments.^6,7^ Moreover, systemic antibiotic treatment often does not provide the right antibiotic concentration to the infected site and has been shown ineffective in chronic granulating wounds.^3,8^ Most importantly, antibiotic resistance is an increasing concern in wound infections, and the Center for Disease Control in the United States is predicting more deaths from antimicrobial infections than from all cancers combined by 2050. Wounds colonized with multiresistant bacteria cause further morbidity to the patient and certainly complicate wound management.^9−11^

There have been numerous attempts to decrease wound infections without resorting to antibiotic use. For example, in recent years, wound dressings with a slow release of silver (Ag) compounds have been marketed and used clinically.^2^ However, apart from the effective role of Ag in killing bacteria, silver ions can be toxic to all microorganisms and cells.^12^ Most other topical antiseptics are also nonselective and may be cytotoxic if used for a longer period of time, thus, significantly hindering the healing process.^13^ Equally as problematic, recent research has also demonstrated that bacteria can develop a resistance to Ag nanoparticles, placing Ag as no better than antibiotics in regard to antimicrobial resistance.^14^

Considering all of the above-mentioned limitations, the management of open skin wound infections will continue to be a challenge for clinicians. Therefore, when it comes to infection, prevention is the first line of defense for every open wound. Thus, preventive wound care should mean managing a wound at an early stage where microbes in a contaminated wound still have limited proliferation and have not yet penetrated deeper into the tissue to evoke a host reaction.^15,16^

In this work, a contact-killing material for the prevention of skin wound infections using antimicrobial peptides (AMPs) is introduced. AMPs are part of the innate immune system with a broad spectrum of activity against microbes.^17^ AMPs work by interacting with the bacterial membrane followed by rapidly rupturing it causing breakdown of the bacterial cell; this mechanism of action is less prone to result in bacterial resistance, making AMPs a better alternative than antibiotics or some nanoparticles (such as Ag).^18−20^

Apart from their antimicrobial effect, AMPs have been shown to have therapeutic potential for the treatment of skin and other epithelial injuries.^21^ Two examples include talactoferrin, which has been shown to stimulate wound healing, and pexiganan, which was developed for the topical treatment of diabetic foot ulcers.^22,23^ Another beneficial factor regarding AMPs in wound management is their target selectivity. This means that unlike most topical antibacterial agents, AMPs do not present any toxicity to host cells when used locally and at the right dosage.^24^

Despite these promising properties of AMPs as a potential alternative to traditional antibiotics and antiseptic agents, the inherent instability of such peptides toward metabolic degradation is an obstacle for bringing them to the clinic.^25^ For example, when exposed to physiological ions or serum conditions, such peptides in their free state have a half-life of only a few minutes to a few hours.^26−28^ One promising way of addressing the poor stability of such peptides and create a long-lasting antibacterial surface is through covalent immobilization of such peptides to wound healing materials.^29^ Several studies have shown that AMPs can retain their antimicrobial activity upon covalent immobilization onto various materials.^30−32^ Moreover, the cytotoxicity of such peptides, which is associated with using high peptide concentrations in a free state, could be avoided by this binding approach.^33,34^

In this work, we developed an antimicrobial hydrogel by covalent immobilization of a cationic AMP, RRPRPRPRPWWWW-NH2 (RRP9W4N), onto ordered amphiphilic mesoporous hydrogels made of cross-linked Pluronic F127 triblock copolymer and water. Such hydrogels were made by cross-linking diacrylated F127 in its liquid crystalline form to give a micellar cubic phase according to its phase diagram.^35,36^ Covalent immobilization of AMPs to such materials formed a soft 3D antibacterial hydrogel with high liquid-absorption properties. It has previously been shown that AMPs physically loaded into dispersed cubic lyotropic liquid crystalline gel particles (cubosomes) exerted high bactericidal activity and improved stability toward enzymatic degradation without releasing the peptides.^37^ We investigated whether the same effects can be observed when AMPs are covalently attached to 3D and macroscopic cubic lyotropic liquid crystalline hydrogels. Such hydrogels possess an amphiphilic structure exposing its hydrophilic sites for the covalent attachment of AMPs, while the hydrophobic parts of the hydrogels provide for hydrophobic interactions with amphiphilic AMPs to increase immobilization and stability of the AMPs.

An ideal wound dressing should do more than just serve as a protective barrier. For example, high absorptive properties and capability to preserve humidity and create a moist environment at the wound site would facilitate wound healing and increase comfort.^38−40^ The wound dressing should be nontoxic, cause no adverse side effects and be easily removable without causing trauma or damage to the wound and surrounding tissue. Finally, they should have antimicrobial properties to prevent infection since infected wounds delay healing and cause patient morbidity. Moreover, the ability to control bleeding in acute wounds is an interesting additional property of a wound dressing. In this study, we investigated whether such criteria are present in AMP-functionalized hydrogels for their future use as an effective antimicrobial wound dressing material. The objective of this study is to evaluate whether covalent immobilization of AMPs onto F127 hydrogels can act as contact-killing surfaces against bacteria and have favorable properties to be used as antimicrobial wound patches.

## Materials and Methods

All chemicals used in this synthesis were provided by Sigma-Aldrich (Stockholm, Sweden) unless specified.

### Manufacturing of Amphiphilic Hydrogels

Diacrylated Pluronic F127 (DA-EO_100_PO_70_EO_100_-DA) (30 wt %) and water (70%) were mixed to form a homogeneous gel possessing a micellar cubic lyotropic liquid crystalline phase according to a well-known phase diagram of the relevant lyotropic liquid crystals.^36^ Irgacure 2959 was added to the gel (2 wt % with respect to the MF127) as a photoinitiator. Mixing was performed in 20 mL glass vials manually using a spatula until a thick and homogeneous gel formed. The gels were spread onto glass slides and kept in a sealed container overnight to set into the micellar cubic phase. The gels were then UV polymerized by a UV LED curing system (UVA, λ = 365 nm, 9 W) for 10 min to form a flexible polymeric hydrogel with a thickness of 1–2 mm. The gels were cut into desired shapes and washed in milli-Q water for 48 h to remove any unwanted byproducts and obtain their fully swollen shape before further analysis and AMP attachment.

### Synthesis of Polymerizable F127 (MF127)

**Figure 1:**
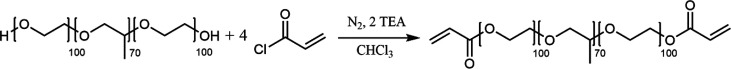
Chemical reaction for the synthesis of the modified Pluronic F127(MF127). TEA stands for triethylamine.

Pluronic F127 was chemically functionalized with polymerizable diacrylate head groups as previously reported.^35,41^ The modified polymer was used for the manufacturing of cross-linked MF127 hydrogels for AMP modification (Figure 1).

### AMP Immobilization on Hydrogels

A solution of antimicrobial peptide (AMP) RRPRPRPRPWWWW-NH2 (RRP9W4N, Red Glead Discovery AB, Lund, Sweden) was prepared in sterilized water to a final concentration of 200 μM. The AMP consists of Arginine-Proline, RP, sequences, which provide high positive net charges for increased electrostatic interactions with bacteria. The hydrophobic part of the peptide consists of Tryptophan, W, which has been shown to improve the peptide interactions with phospholipid membranes and increase bactericidal activity.^26,42^ Covalently attached RRP9W4N has previously been shown to possess high antibacterial activity against various bacterial strains and improved stability in physiological salt and serum conditions.^43^

For the covalent attachment of AMP to the hydrogels, prior to AMP modification, the clean hydrogels were submerged into a solution of 1-ethyl-3-(3-(dimethylamino)propyl) carbodiimide hydrochloride (EDC) and *N*-hydroxysuccinimide (NHS) mixed in MES buffer (pH 6) at a final concentration of 2 mg/mL and were allowed to react for 30 min on a slow shaker at room temperature. Hydrogels were then washed three times in PBS (pH 7.4), sterilized using 70% ethanol, and suspended in 1 mL of a 200 μM AMP solution in sterilized water for 2 h at RT. The surfaces were washed three times with sterile water to remove unreacted peptides. We measured the maximum number of covalently attached AMPs before the washing steps to be 0.031 ± 0.013 mg/disc (4 mm in diameter and 0.7 mm in thickness) using UV–visible spectroscopy at λ = 280 nm. For this measurement, we used pure PBS as the blank and measured the AMP concentration solution after the immobilization process using a standard curve.

**Figure 2:**
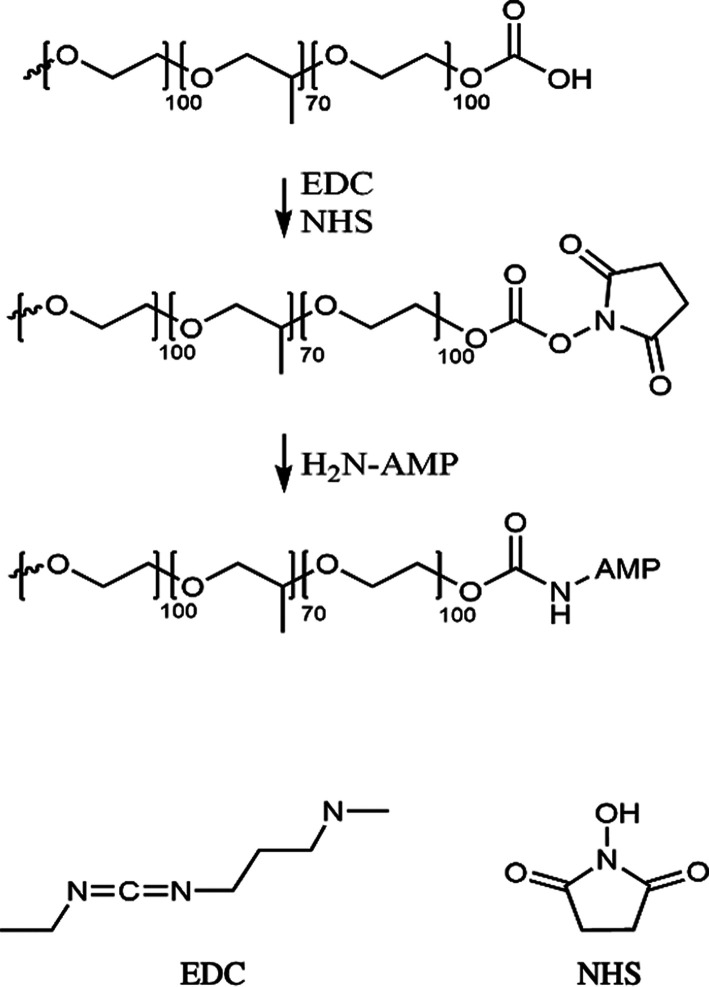
Schematic of EDC-NHS activation and AMP attachment on hydrogels.

All AMP and control hydrogels were used in experiments within 7 days from production. A schematic of AMP covalent immobilization via EDC-NHS activation is shown in Figure 2. This method utilizes carboxyl groups present on the surface of the hydrogels for direct coupling with primary amine groups present in the AMP. EDC was used to activate carboxyl groups, and NHS was used to improve the reaction efficacy and dry state stability of the reactive intermediates.

### Bacterial Culture and Growth

*Staphylococcus epidermidis* (ATCC 35984), *Staphylococcus aureus* (CCUG 56489), and *Pseudomonas aeruginosa* (CCUG 10778) were used to assess colony formation on the hydrogels. One day prior to the experiments, a fire-sterilized metal loop was used to withdraw a single colony from cultured brain heart infusion (BHI) agar plates of each strain to inoculate a tube of 5 mL of tryptic soy broth (TSB), which was cultured at 37 °C for 6 h under shaking at 250 rpm, further diluted in TSB (1:1000), and cultured at 37 °C under shaking at 150 rpm overnight to reach the stationary phase for bacterial growth.

The optical density (OD) of the bacterial culture was adjusted to 0.7 at 600 nm, estimated to give 10^9^ colony-forming units (CFU/mL) using a spectrophotometer. The bacterial suspension was centrifuged for 10 min at 2500 rpm, and the bacterial cell pellet was suspended in fresh TSB medium. Approximately 10^9^ CFU in 1 mL of suspension was seeded onto hydrogels (with and without AMP modification) placed in 24-well plates. Bacteria were then cultured for 24 h under static culture conditions at 37 °C to promote biofilm formation on the surfaces. Finally, the hydrogels were rinsed three times with fresh PBS to wash off the unattached planktonic bacteria before biofilm imaging with fluorescent microscopy.

### MRSA and MDR **Escherichia coli** Culture and Growth

A single bacterial colony of MRSA (ATCC 43300) and MDR *E. coli* (ATCC BAA-2471) was isolated from cultured BHI agar plates and inoculated in 5 mL of TSB cultured in a shaking incubator at 37 °C overnight. The optical density of the bacterial suspension was adjusted to OD_620 nm_ = 0.52 (equivalent to 10^9^ CFU/mL) and further diluted in TSB to obtain an inoculum of 10^6^ CFU/mL. Material samples were placed in a standard 24-well plate, and 1 mL of inoculum was used to seed each sample followed by incubation for 24 h at 37 °C and 5% CO_2_.

### Bacterial Live/Dead Fluorescence Analysis

To directly analyze the population of live and dead cells of the different bacterial species on the surface of the hydrogels, fluorescent microscopy with live/dead staining was performed. The live/dead population of *S. epidermidis, S. aureus*, and *P. aeruginosa* was assessed in a Zeiss Axio Imager Z2m fluorescent microscope, whereas a Zeiss Axio Observer was used for the analysis of MRSA and MDR *E. coli* on the hydrogel surfaces.

After incubation, the bacterial suspension was removed from the hydrogels and the samples were gently rinsed twice with PBS. A drop of live/dead staining solution from LIVE/DEAD BacLight Bacterial Viability Kit L7007, prepared according to the manufacturer’s instructions, was placed on top of the sample to cover its surface. The samples were incubated for 10 min at room temperature in a dark environment before imaging. Prior to imaging, each sample was divided into 20 equal parts to prespecify the imaging spots. Twenty images from each surface were used to obtain the image analysis data using ImageJ software. All live/dead-experiments were conducted three times with three replicates.

### Stability Assessment

#### Serum Stability

The duration of the antimicrobial functionality of the AMP-hydrogels was evaluated upon incubation in human serum. After AMP modification, the hydrogels were placed in wells of a 24-well microtiter plate. To each well, 0.4 mL of 20% human serum (from human male plasma) (Sigma) diluted in sterile milli-Q water was added and samples were incubated at room temperature from 1 h to 2 days. At each time point the serum was removed; the hydrogels were washed in PBS, and bacterial culturing using 10^8^ CFU/mL of *S. aureus* (CCUG 56489) suspension in TSB for 24 h was performed to investigate how long the attached AMPs retained their bactericidal activity. The experiment was carried out with sample duplicates, and two independent experiments were performed (*n* = 2).

#### Zone of Inhibition

In order to confirm that the AMPs were covalently attached to the hydrogels, preventing them from leaking out, zone of inhibition tests were performed. The *S. aureus* (CCUG 56489) culture was made in TSB at an optical density between 0.55 and 0.7. The bacteria pellet was collected by centrifugation (2500 rpm, 10 min) and suspended in 20 mL of fresh TSB, yielding 10^9^ CFU/mL. BHI agar plates were then streaked evenly with 100 μL of the bacterial suspension to reach confluent growth and cover the entire agar surface. The hydrogels (two with and two without AMP) were placed on top of the agar plates. As a control, hydrogels were submerged in the same solution of AMP (200 μM) without the EDC coupling agent, rinsed in PBS, and used to show the leakage difference between AMP covalent and physical attachment. The plates were incubated at 37 °C overnight. Afterward, the inhibition zones around the hydrogels where no bacteria had grown were measured from a digital photograph and compared between the samples. The inhibition zone area outside of the hydrogel contours was considered a consequence of leaked AMP from the hydrogels into the surrounding agar.

#### PBS Washing

To assess the stability of covalently bonded AMPs onto hydrogel surfaces, a 14 day washing experiment with PBS was performed. In this test, hydrogel with covalently bonded AMPs and physically absorbed AMPs (submerged in 200 μM AMP solution without the EDC/NHS coupling) as controls were placed in wells of a 24-well microtiter plate. To each well, 1 mL of PBS (pH = 7) was added, and samples were incubated at room temperature for up to 14 days. Samples were taken out at the end of each time point (each day, 0–14 days) and bacterial tests using 10^8^ CFU/mL of *S. epidermidis* (ATCC 35984) suspension in TSB for 24 h was performed to investigate how long each surface could retain their antibacterial activity. The experiment was carried out with sample triplicates, and two independent experiments were performed (*n* = 2).

### Blood Coagulation Test (Platelet Count)

Blood collecting Eppendorf tubes, pipet tips, and tubings to draw blood were heparinized to avoid unwanted blood activation. Heparinization was performed according to the Corline method (Corline Biomedical AB, Uppsala, Sweden): a layer-by-layer assembly method with alternating incubation with a polymeric amine and a heparin conjugate to obtain a double-coated heparin layer.

Fresh blood from two healthy human volunteers was collected in heparinized tubes containing 1 IU/mL of a heparin solution (Leo Pharma A/S, Ballerup, Denmark). The blood was used fresh after sampling. In an Eppendorf tube, 1 mL of blood with 4 mM EDTA was collected for use as a reference point (referred herein as initial).

Samples were conditioned by adding 1 mL of PBS and vortexed at 600 rpm for 30 min prior to the experiment. The hydrogels (control and AMP modified) were placed in heparinized 2.5 mL Eppendorf test tubes. A volume of 100 μL of PBS was added to soak the samples, and subsequently 1 mL of fresh blood was added to each tube. The tubes were then rotated on an orbital shaker (Incubating Waver, VWR) for 60 min at 37 °C. As blank controls, 1 mL of blood was added to an Eppendorf tube without any hydrogels and treated with the same conditions. After the experiments, the blood was carefully collected from the tubes and mixed with EDTA, giving a final concentration of 4 mM. The number of platelets was counted using a Sysmex XP-300 hematology analyzer (Kobe Japan). Samples were run in duplicate with blood from each donor (*n* = 2). Ethical approval was obtained from the regional ethics committee Dnr: 2008/264.

### MTT Assays

Primary fibroblasts (Gibco Human Dermal Fibroblasts, adult (HDFa); Fisher Scientific) were thawed and subcultured according to the supplier’s recommendation. Briefly, cells were cultured and expanded in Dulbecco’s Modified Eagle Medium (DMEM) cell media supplemented with 1 μg/mL hydrocortisone, 10 ng/mL human epidermal growth factor, 3 ng/mL basic fibroblast growth factor, 10 μg/mL heparin, 10 μg/mL gentamicin, and 0.25 μg/mL amphotericin B and 10% FBS by volume at 37 °C and 5% atmospheric CO_2_. To perform MTT assays, hydrogel samples (thin hydrogel discs punched out with a 4 mm biopsy punch) were soaked in 1 mL of supplemented DMEM for 3 days. A volume of 200 μL of the sample-exposed media was added to the wells of a 96-well plate. As negative controls, media not exposed to any samples were used. Four replicates of each kind were used, and the experiment was run twice (*n* = 2). The fibroblast concentration was calculated using a Bürker counting chamber. Five thousand fibroblasts were then added to each well. The plate was incubated at 37 °C for 3 days. After incubation, the media was aspirated from each well and replaced with 100 μL of fresh media (of corresponding type) to which 10 μL of the MTT solution (5 g of MTT in 1 mL of PBS) was added and mixed gently by pipetting. After 4 h additional incubation, 100 μL of an SDS solution (1 g of SDS in 10 mL of 0.01 M HCl) was added to each well and placed in the incubator for another 4 h. The absorbance of each well was read at 570 nm by a spectrophotometer (Thermo Scientific Multiskan GO). The blank control (media and MTT stain without any cells) absorbance was subtracted from each value, and the viability of the cells was calculated by dividing the absorbance values with the mean of the negative controls. A cell viability value of 70% was considered as a standard for nontoxic material sample.

### Pilot In Vivo Infection Study Using a Rat Model

#### Animal Handling

Seven female Sprague–Dawley rats (200–300 g) fed on a standard pellet diet and water were used in the study, which was approved by the Local Ethical Committee for Laboratory Animals (Dnr 1091/17). The animals were housed together (2–3 rats/cage) and kept at the infection unit at the animal facility with daily supervision. Anesthesia was induced by isoflurane inhalation (4% with air flow of 650 mL/min) and maintained with continuous administration of isoflurane (∼2% with an air flow of 450 mL/min) via a mask (Univentor 400 anesthesia unit, Univentor, Zejtun, Malta). The back of the rats was shaved and cleaned with chlorhexidine (5 mg/mL; Fresenius Kabi, Norway). On the back of the rats, six separate incisions were made and pockets created by blunt dissection in the soft tissue under the skin into which hydrogel disks were inserted.

#### Surgical Procedure

On the back of each rat, three control hydrogels and three AMP-hydrogels were inserted. A volume of 50 μL of saline with or without *S. aureus* (ATCC 25923), at two different dosages (10^4^ and 2 × 10^6^ CFU), was pipetted into the pocket before careful closure of the wound with intracutaneous sutures followed by 2–3 simple sutures (Ethilon 5-0 FS-2, Eticon, Johnson & Johnson, Scotland). The back was cleaned with saline, and each rat received analgesics at a dose of 0.03 mg/kg (Temgesic, Reckitt Benckiser, UK).

Animals were sacrificed after 24 or 72 h with an overdose of pentobarbital (60 g/L, APL, Sweden) after a short anesthetic induction with isoflurane. The back of the rats was cleaned with chlorhexidine, and all sutures were removed. The implants were retrieved into a saline-containing tube, and the exudates were obtained from the pockets by repeated aspiration (5×) with 500 mL of phosphate-buffered saline (PBS) and kept on ice until CFU determination.

### Statistical Analysis

The numerical data for the live/dead bacterial cells were obtained by calculating the red and green fractions from fluorescent microscopy images using ImageJ software and are presented by their mean values and standard deviations. The experiments were conducted three times (*n* = 3) with three sample replicates. For the serum stability test, duplicate samples were used, and two independent experiments were performed (*n* = 2). Twenty images from each surface were used to obtain the image analysis data. Paired Student’s *t* test was performed for data analysis. Data from MTT assay are presented by mean values and standard deviation, averaged over four replicates and two biological replicates (*n* = 2). Data analysis was performed using Welch’s *t* test. Data from the blood coagulation test are presented as mean values and standard deviation, with four replicate samples using blood from two different donors (*n* = 2). A *p*-value < 0.05 was considered significant.

## Results and Discussion

### Manufacturing of Amphiphilic Hydrogels

In this study, a hydrogel consisting of a cross-linked diacrylate-modified triblock copolymer (F127) and water was used as a substrate for AMPs functionalization. The material is an antimicrobial hydrogel based on the covalent immobilization of antimicrobial peptides (AMPs) onto F127 hydrogels. As a preventive wound care patch, such materials here demonstrated local killing of bacteria in contact with the patch surface, showing potential to decrease the bioburden (i.e., the amount of pathogen) in the wound and prevent bacteria invasion deeper into the tissue.

**Figure 3:**
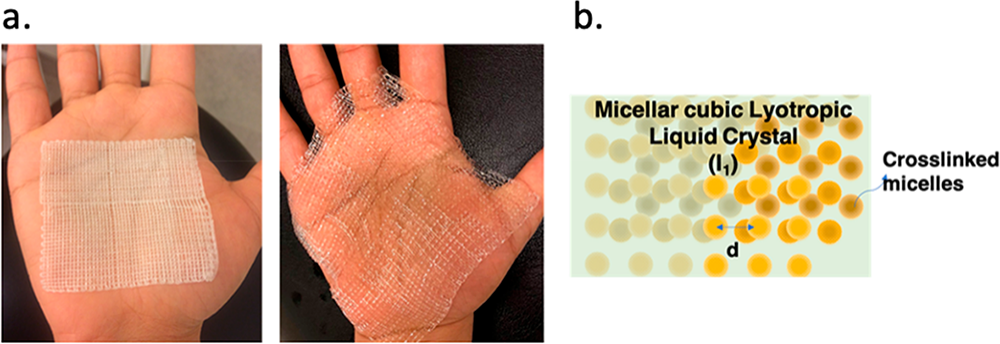
(a) Three-dimensional-printed hydrogel mesh before (left) and after (right) swelling in water. (b) Schematic of the micellar cubic lyotropic LLC phase which is the mesostructured phase of the hydrogel used in this work. (l1) indicates micellar cubic phase.

The hydrogel possessed an ordered mesoporous structure consisting of alternating hydrophilic and hydrophobic domains previously characterized using small-angle X-ray scattering (SAXS).^44^ The hydrogel retained its ordered structure after cross-linking.^35,41^ This ordering repeats itself throughout the gel, exposing both hydrophilic and hydrophobic domains on its surface and making them available for the covalent attachment of peptides. On the basis of earlier observations that physically immobilized (not covalently anchored) AMPs on hydrogel cubosomes possessed high antimicrobial activity,^37^ it was speculated that the amphiphilicity of the hydrogels will result in favorable hydrophobic interactions with the AMPs, which also are amphiphilic. This interaction was hypothesized to result in the orientation, alignment, and specific AMP binding (on the hydrophilic or hydrophobic parts of the hydrogel). Moreover, the hydrogel has a high liquid absorption capacity and owing to its amphiphilicity can maintain both polar and nonpolar liquids in its structure. In Figure 3a, a 3D-printed hydrogel mesh is shown before and after water absorption. A schematic of the mesostructured phase of the hydrogel is shown in Figure 3b.

### Antimicrobial Peptide Modification and Bacterial Response

**Figure 4:**
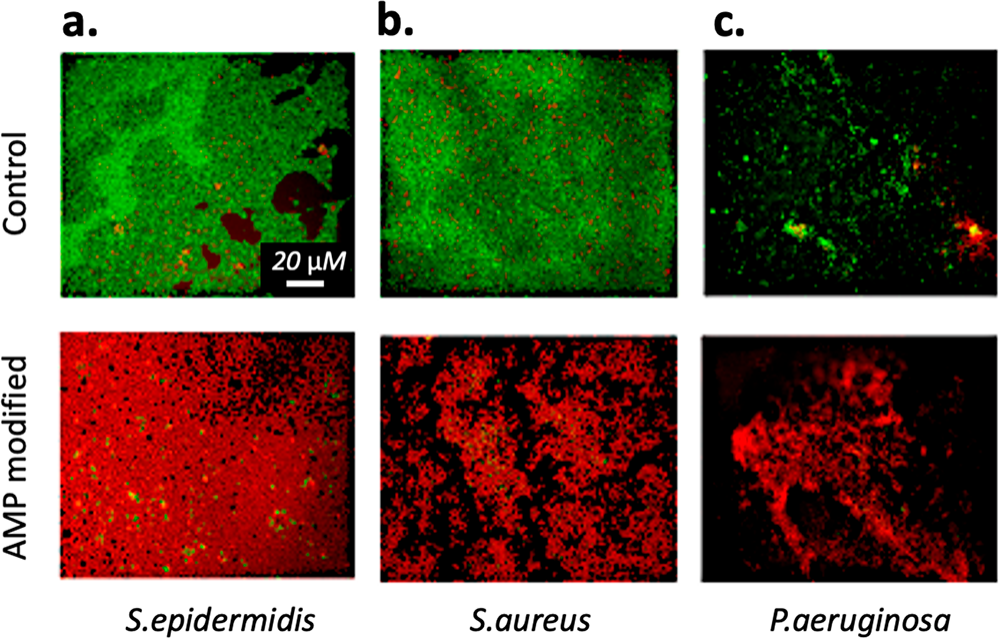
Images of bacteria attached onto hydrogels with and without AMP. Bacteria were stained by SYTO 9 and propidium iodide. Live bacteria appear as green, and dead bacteria appear as red. (a) *S. epidermidis*, (b) *S. aureus*, and (c) *P. aeruginosa.*.

**Figure 5:**
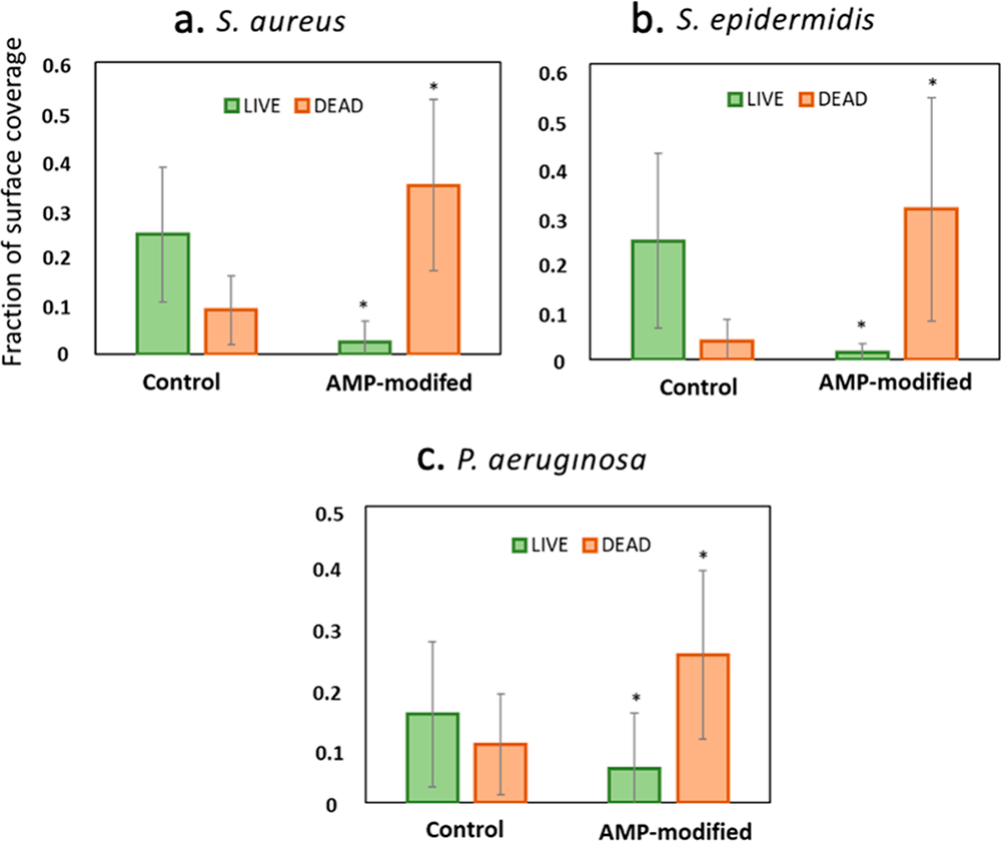
Quantitative analysis of the live and dead fractions of (a) *S. aureus*, (b) *S. epidermidis*, and (c) *P. aeruginosa* colonization on hydrogel surfaces with and without AMP attachment. Values were obtained from analyzing fluorescent images using ImageJ software. Values are mean ± SD, triplicate samples were used, and three independent experiments were performed (*n* = 3), *p < 0.05 compared to control groups (hydrogels with no AMP).

The results of this study showed that the AMP-hydrogel had high antimicrobial activity against *S. epidermidis*, *S. aureus,* and *P. aeruginosa*. In Figure 4a, 4b, and 4c, images from fluorescent microscopy with live/dead staining show a very high bactericidal efficiency of the AMP-hydrogel compared to the control hydrogel. Moreover, the total colonized area by live bacteria on the surfaces of AMP-hydrogels was significantly smaller than on the control hydrogel. As seen in Figure 5a and 5b, a ∼90% reduction of live *S. epidermidis* and *S. aureus* and in Figure 5c a ∼70% reduction of live *P. aeruginosa* were observed on the AMP-hydrogel surfaces compared with control hydrogels. In addition, the proportion of dead cells increased by ∼70% for *S. epidermidis*, ∼80% for *S. aureus*, and ∼50% for *P. aeruginosa.*

**Figure 6:**
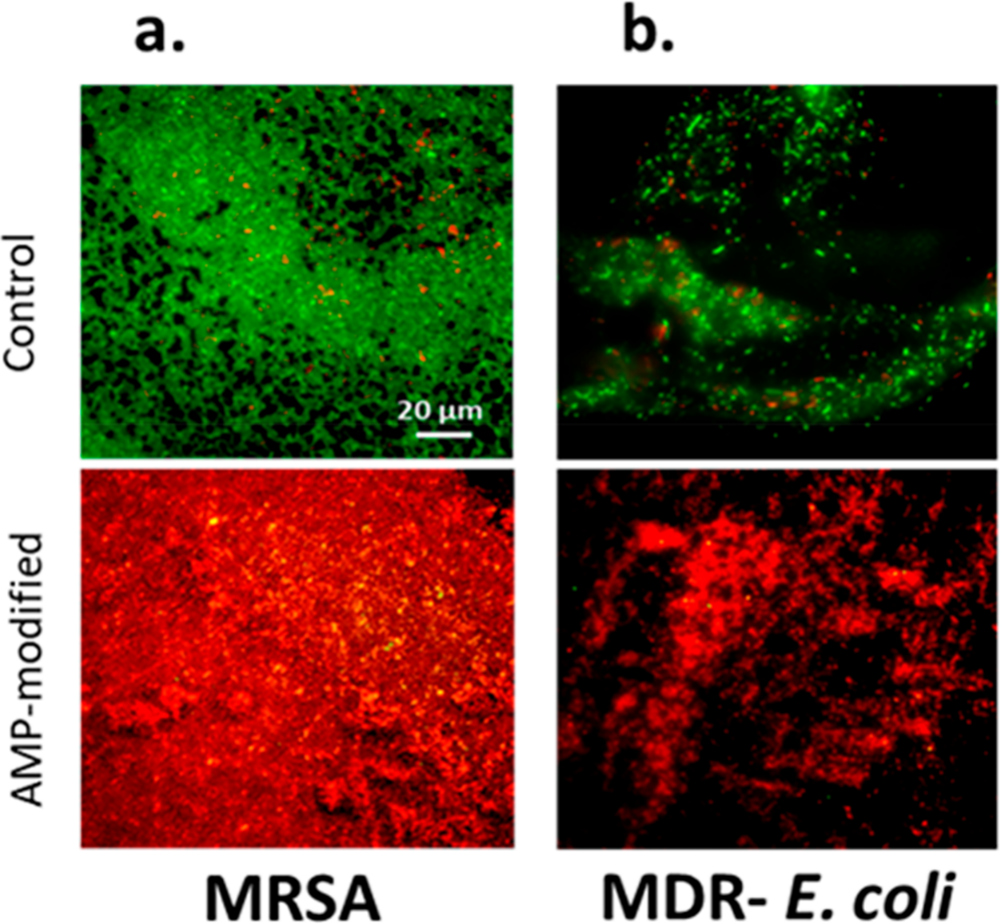
Fluorescence images of bacterial cells attached onto hydrogels with and without AMP. Bacteria were stained by SYTO 9 and propidium iodide. Live bacteria appear as green, and dead bacteria appear as red. (a) Methicillin-resistant *S. aureus* and (b) multidrug-resistant *E. coli*.

**Figure 7:**
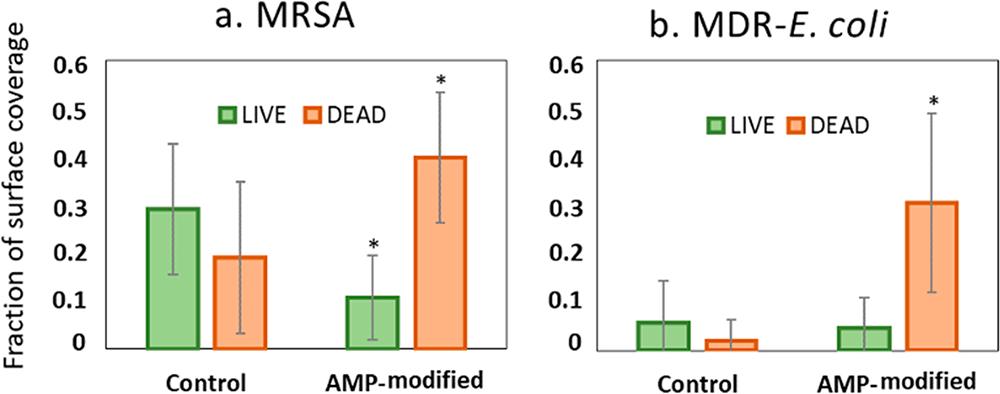
Quantitative analysis of the live and dead fractions of antimicrobial resistant bacteria. (a) Methicillin-resistant *S. aureus*, and (b) multidrug-resistant *E. coli* on hydrogel surfaces with and without AMP attachment. Values are mean ± SD, triplicate samples were used, and three independent experiments were performed (*n* = 3), *p < 0.05 compared to control groups (hydrogels with no AMP).

Similarly, the AMP-hydrogels were effective at decreasing colonization by antimicrobial-resistant strains, such as MRSA and MDR *E. coli*. Specifically, the images of live/dead bacteria obtained from fluorescent microscopy (Figure 6a and 6b) show a greater population of dead cells on the AMP-hydrogels compared to control hydrogels. According to Figure 7a, the results showed a ∼65% reduction in the fraction of live and a ∼50% increase in the fraction of dead MRSA on the AMP-hydrogels. While, as shown in Figure 7b, the fraction of dead MDR-*E. coli* had a ∼90% reduction on AMP-hydrogels, there was no considerable decrease in the fraction of live cells.

Overall, the results from the live/dead fluorescence *in vitro* study suggest that covalent attachment of AMPs onto the amphiphilic hydrogel formed a very effective contact-killing effect toward a broad spectrum of bacterial species (including multiresistant strains). If such materials are to be used as antibacterial wound patches, they can potentially kill a range of Gram-positive and Gram-negative bacteria present at the wound surface and may hinder their penetration into deeper tissues.

### Stability Assessment

#### Serum Stability

**Figure 8:**
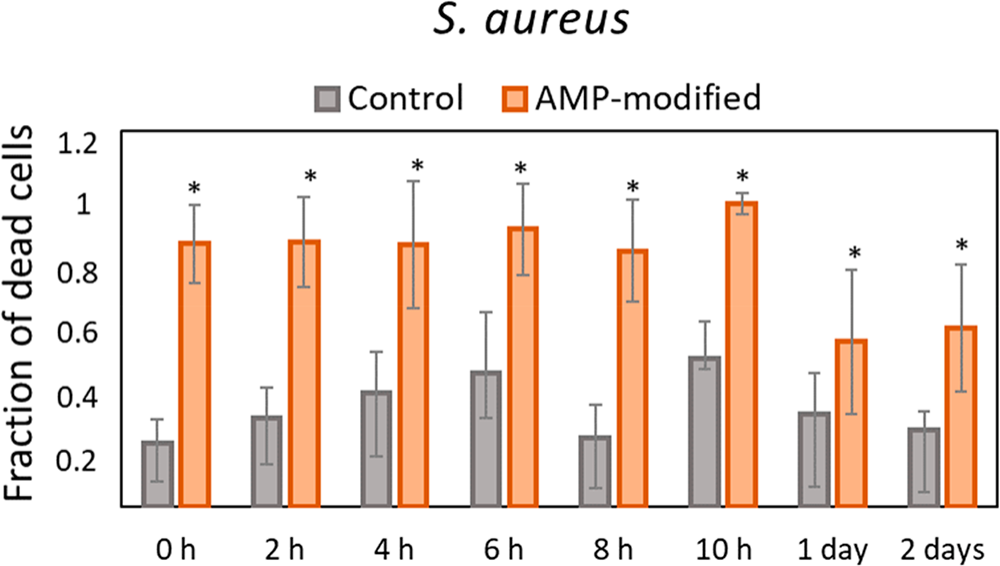
Proportion of dead cells from *S. aureus* after 24 h on hydrogel surfaces with and without AMP and previously incubated in 20% human serum for up to 2 days. Data is calculated from the images of live/dead stained bacteria at each time point. Values are mean ± SD, duplicate samples were used, and two independent experiments were performed (*n* = 2), *p < 0.05 compared to control groups (hydrogels without AMP) at each time point.

In the present study, the stability of peptides was examined in human blood serum. The stability of peptides in serum or plasma is considered as a representative model for their in vivo stability.^45^ As seen in Figure 8, the covalently attached peptides kept their antibacterial effect against *S. aureus* for up to 2 days, showing a significantly higher proportion of dead cells on AMP samples compared to control samples for each time point when incubated in human serum, while the serum stability of the same analogues of the peptide in diluted serum showed a half-life between 0.5 and 6.5 h in previous studies.^26^ The results in the present study suggest that covalent attachment of peptides can considerably increase peptide stability compared to when the peptides are released. Previous work has shown that the AMP under investigation also possessed an increased stability after 24 h when covalently bonded to an elastin-like polypeptide matrix.^43^

Suggested explanations for why AMPs lose their activity in serum are either the increased ionic strength or the presence of other proteins and enzymes in the serum. Peptides rich in cationic components are more susceptible to degradation since the charged components in their structure, such as arginine and lysine, expose more cleavage sites to enzymes present in blood.^26^

#### Zone of Inhibition

**Figure 9:**
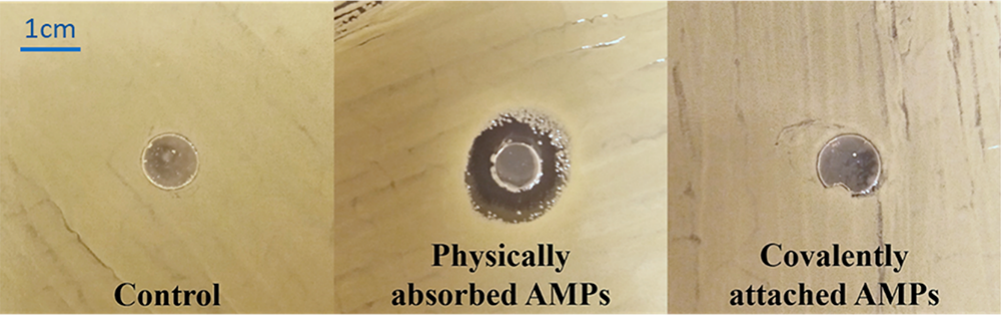
Images of the zone of inhibition of *S. aureus* (CCUG 56489) around three different types of hydrogels: from left to right is the control hydrogel, physically adsorbed AMP-hydrogel, and covalently bonded AMP hydrogels.

In order to ensure that the AMPs are covalently attached to the hydrogels and do not leak out, a zone inhibition test was performed. Hydrogels with covalently attached AMPs were compared to hydrogels with physically adsorbed AMPs. Hydrogels were placed on top of a BHI agar plate previously streaked with *S. aureus* and incubated at 37 °C overnight. Images of the zone inhibition tests are presented in Figure 9. A clearly visible inhibition zone (radius of ∼7 mm) around the hydrogels with physically adsorbed AMPs could be measured, whereas no inhibition zones were present around the hydrogels with covalently bonded AMPs nor control hydrogels. The visible inhibition zone is a result of AMPs being released from the hydrogels, thus killing bacteria also further away from the surface, whereas a lack of inhibition zone implies that the covalently bonded AMPs did not leach out from the hydrogels. These observations correlated well with the results from the microscopy images of fluorescent-tagged AMPs (5(6) carboxyfluorescein-AMP) covalently bonded and physically adsorbed onto hydrogels and washed for 3 weeks (Figure S1, Supporting Information). It was shown that AMPs that were covalently bonded onto hydrogels did not leak out upon washing with milli-Q water for up to 3 weeks.

#### PBS Washing

**Figure 10:**
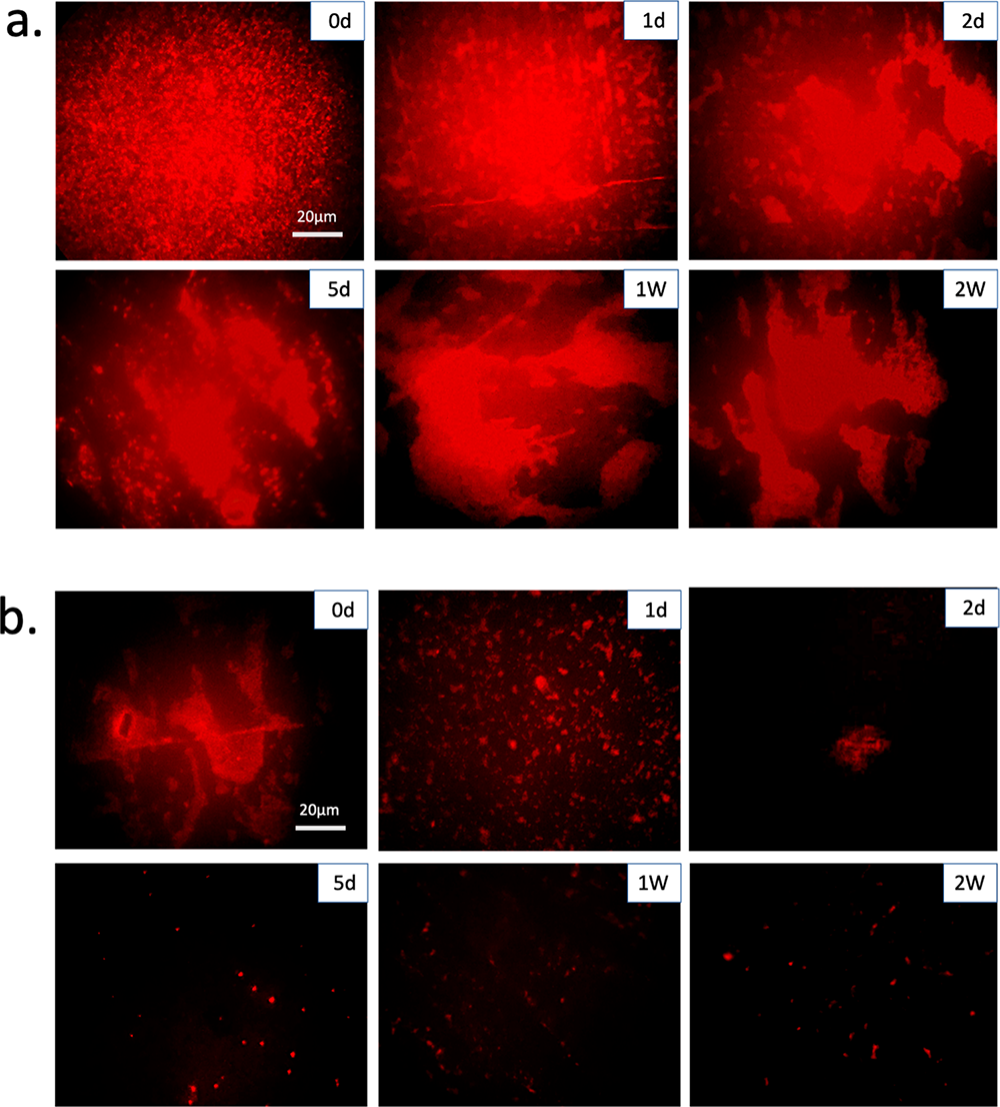
Fluorescence images of dead bacterial cells attached onto hydrogels incubated in PBS up to 2 weeks. (a) Covalently bonded AMPs using EDC/NHS coupling. (b) Physically absorbed AMPs. Dead bacteria were stained by propidium iodide. d = days and W = weeks.

To assess the long-term stability of covalently bonded AMPs using EDC/NHS chemistry compared to when they are only physically absorbed onto hydrogel surfaces, a PBS washing test was performed for a duration of 2 weeks. The results showed that the covalently bonded AMPs could retain their antibacterial activity against *S.epidermidis* for 2 weeks as shown in Figure 10a, while the physically absorbed AMPs lost their activity already after 1 day (Figure 10b). These results clearly show that covalent immobilization of AMPs is vital for creating AMP-modified hydrogels that preserve their activity for a long term without leaching AMPs onto the surroundings.

### Pilot In Vivo Tests in a Rat Model

**Figure 11:**
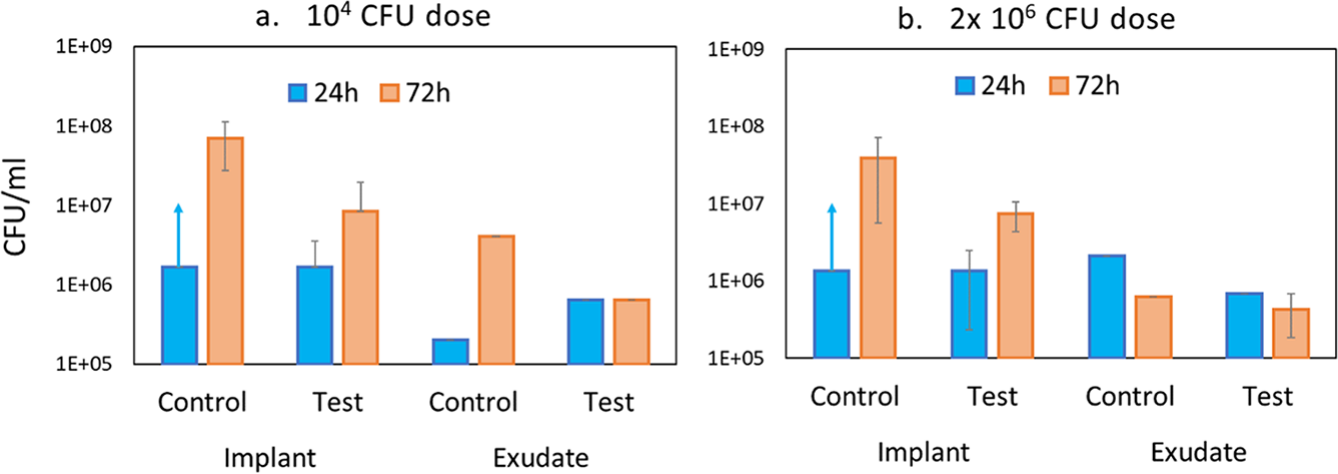
Colony-forming units of *S. aureus* (ATCC 25923) associated with hydrogel implants and present in exudates surrounding the hydrogel at 24 and 72 h after inoculation with (a) 10^4^ and (b) 2 × 10^6^ CFU of *S. aureus*. Test = AMP-hydrogel; Control = hydrogel without AMPs. Note: Control hydrogel at 24 h with both infectious doses: arrow represents a “noncountable CFU” due to overgrowth of *S. aureus*. CFU/mL for these samples is estimated to be higher than the corresponding Test hydrogel at 24 h).

A pilot study was performed to assess the antibacterial activity and performance of the AMP-hydrogels in vivo using a rat model previously used for the evaluation of biomaterial-associated infection and inflammation around titanium surfaces.^46^ Two dosages of bacterial inoculation (10^4^ and 2 × 10^6^ CFU) were used, and the number of viable CFU counts was obtained from retrieved implants and exudates at two different time points (24 and 72 h). The CFU results are presented in Figure 11 and indicated 10–100× less viable counts of *S. aureus* on AMP-hydrogels compared with control hydrogels at both time points and with both inoculums (Figure 11a and 11b). Although the difference between the two hydrogels decreased over time, there was still approximately 10× less bacteria on AMP-hydrogel disks than on control disks after 72 h. Over time, there was a clear increase in CFU on the AMP-hydrogels, indicating reduced antibacterial efficiency. Such behavior can be explained with our results on AMP stability in serum, which showed that the AMPs have lost approximately 50% of their antibacterial functionality after 48 h in human blood serum. Hence, it is likely that the AMPs lost even more of their activity after 72 h in the *in vivo* environment. The number of viable bacteria in the surrounding exudate was more stable over time, and the results did not follow a regular pattern. This is likely a result of a very local antibacterial effect around the hydrogels due to the covalent bonding of the AMP to the hydrogel surface. Importantly, the control rats without inoculated bacteria did not show any *S. aureus* growth.

Overall, the results from the retrieved hydrogels in the *in vivo* pilot study showed a decrease in the number of *S. aureus* adhered to AMP-hydrogels compared to control hydrogels. However, an additional study using an infected wound model is needed to confirm if the AMP-hydrogels can keep their activity.

### Blood Coagulation Test

**Figure 12:**
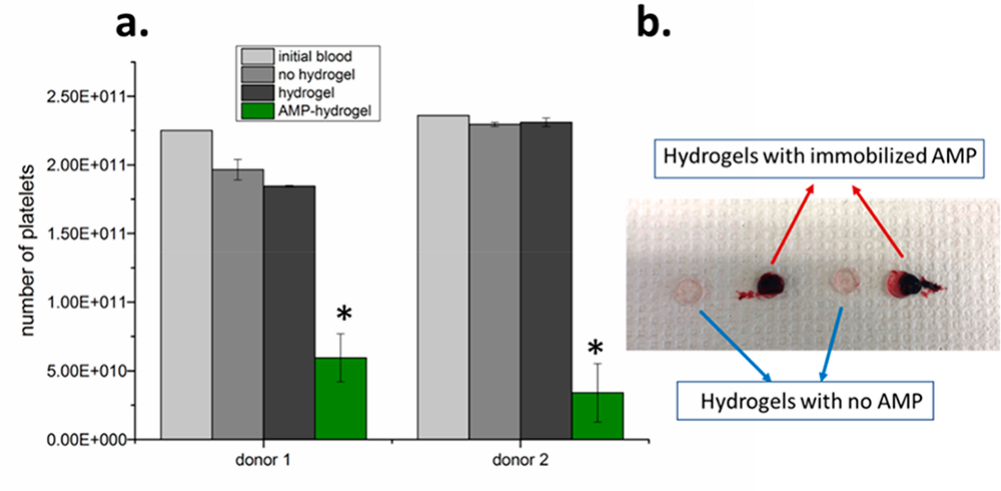
(a) Number of platelets after whole blood incubation at 37 °C for 60 min. Data represents mean ± SD of four replicate samples, *p < 0.05 compared to control groups. (b) Photograph showing the appearance of the hydrogels after 1 h of blood contact.

One key requirement for proper wound management is to control bleeding. Blood coagulation involves the formation of a fibrin clot in the presence of the wound, which can trap the platelets and release inflammatory chemokines. Blood coagulation initiates lateral responses to activate inflammatory responses and subsequent wound healing.^47,48^ Therefore, it was investigated how the presence of AMPs on the hydrogels affect blood coagulation. Here, a whole blood test using fresh blood from two donors was performed, and the number of platelets before and after 1 h of exposure to blood was quantified. A large visible clot was observed on AMP-hydrogels, whereas no clot formation was observed on the control, as shown in Figure 12b. In line with the visual inspection results, platelet counts/counts of free platelets in the blood (shown in Figure 12a) were significantly lower in the presence of AMPs. The capacity of blood to clot upon contact with AMP-hydrogels can be explained by the interaction of positively charged AMPs with blood cells, platelets, and plasma fibronectin to induce hemostasis. Furthermore, the capability of hydrogels to absorb blood facilitated the interaction of blood cells with AMPs to induce blood clotting.^49,50^ The results suggest that using AMP-hydrogel as a wound patch can accelerate blood coagulation and clot formation for a bleeding wound. That can be considered as a desirable additional property provided by AMP-hydrogels apart from the antibacterial effect.

### MTT Assays

**Figure 13:**
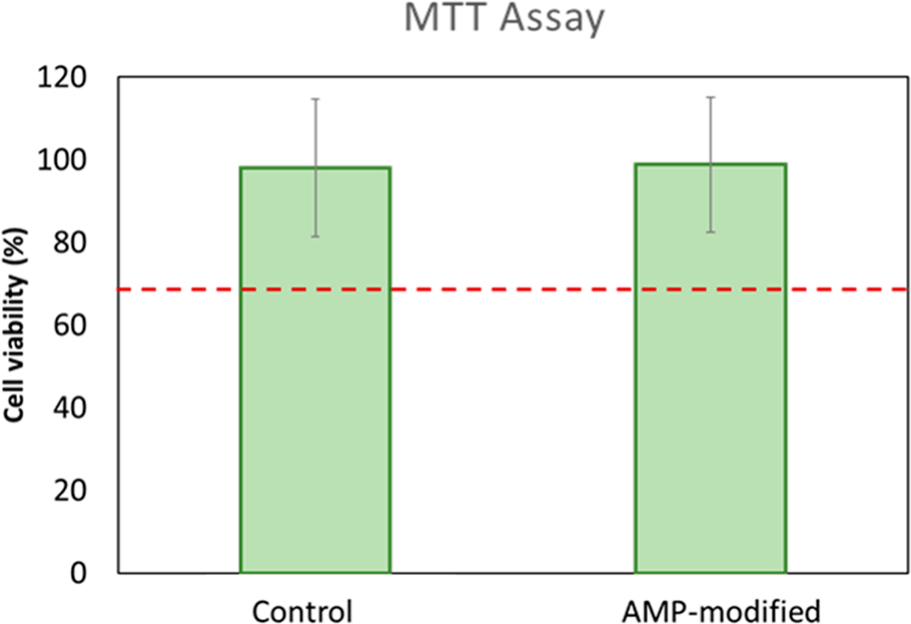
Fibroblast viability when subjected to hydrogel-exposed media, with or without AMP, as measured by MTT assays. Red dotted line indicates 70% cell viability. Values are mean ± SD, four replicates of each sample type were used, and the experiment was run twice (*n* = 2).

The viability of primary fibroblasts exposed to hydrogel-conditioned media, with or without AMP-modification, was studied by MTT assays. The results are shown in Figure 13, where the dotted red line indicates 70% cell viability, which according to the international standard ISO 10993-5:2009 implies a nontoxic material. A value of 100% is attributed to cell growth for negative controls (no exposure to hydrogels). As observed, none of the hydrogel-exposed media was toxic to human fibroblast cells according to MTT assays. The results suggest that while the presence of AMPs onto a hydrogel introduces a strong bactericidal effect, there were no leachable substances from the AMP-hydrogel that can induce toxicity to the human-derived cells used in this study. Such a property makes AMP-hydrogels a suitable antibacterial surface to be used for the prevention of bacterial infection in wound management without harming healthy tissue cells behavior.

### AMP-Hydrogels in Wound Care Applications

The AMP-hydrogel designed and evaluated in this study offers suitable properties of being an ideal wound dressing for the prevention of infection and acceleration of wound healing. F127 hydrogels have high water content in their structure, which facilitates a moist environment, which is often a primary requirement for a wound dressing material. Moreover, their high absorptive properties imply that they can absorb large amounts of liquids, a property that is crucial for moisture exchange activities between the wound bed and the material to promote healing.^51^ It is also known that a high moisture content and softness of the hydrogels can have a soothing effect, reduce pain, and increase comfort in the wound area as compared to viscous fibers and cotton.^52^ Hydrogels in general are nonadhesive, and some can even be similar to living tissues in terms of their biocompatibility properties.^53,54^ In addition, hydrogels are nontoxic and easily processable into different shapes to fit different types of wounds.

Infected wounds delay the healing process. As shown here, AMP-hydrogels have an ability to kill a broad range of bacterial species including antibiotic-resistant strains, representing a preventive wound dressing that can decrease microbial bioburden and risk of infection in the wound.

Further, the AMP-hydrogel was shown to accelerate blood clot formation in contact with blood, which is yet another important criterium for wound dressing materials. As blood interacts with the AMP-hydrogel, it activates platelets and forms a fibrin plug to prevent excessive blood loss and to evoke an inflammatory response, which is beneficial for wound healing to take place.

To further assess the antibacterial performance of AMP-hydrogels, additional investigations including a negative AMP scramble sequence and the use of plasma (which contains fibrinogen as a key clotting protein) in the in vitro experiments on stability as well as an *in vivo* wound healing model are required.

## Conclusion

Here, we developed an amphiphilic antibacterial hydrogel with the potential to prevent skin wound infections by covalently binding a positively charged AMP, RRP9W4N, to an ordered mesoporous hydrogel consisting of a cross-linked lyotropic liquid crystal. The material showed a broad spectrum of antibacterial activity against Gram-positive, Gram-negative, and antibiotic-resistant bacteria and did not release any toxic leachable substances to human fibroblast cells. The stability of AMPs in human serum increased due to the covalent bonding to the hydrogel and more than 50% of its antibacterial activity was retained for up to 48 h. The AMP-hydrogel developed in this work introduces a new contact-killing material for decreasing the bacterial bioburden in open wounds. This approach introduces new promise for AMPs to be used in wound management applications to decrease unnecessary antibiotic usage.

## Supporting Information Available

Results from the microscopy images of fluorescent-tagged AMPs (5(6) carboxyfluorescein-AMP) covalently bonded and physically adsorbed onto hydrogels. (PDF)
