# Supplementary material for: Antimicrobial Peptide-Functionalized Mesoporous Hydrogels
Source: ACS Biomater Sci Eng. 2021 Mar 15;7(4):1693–702. doi: 10.1021/acsbiomaterials.1c00029 (PMC8153390; doi:10.1021/acsbiomaterials.1c00029)
Supplement: Supplementary file 1 — ab1c00029_si_001.pdf [file ab1c00029_si_001.pdf]

Antimicrobial Peptide Functionalized Mesoporous Hydrogels

Saba Atefyekta[1a], Edvin Blomstrand[1a], Anand K. Rajasekharan[1], Sara Svensson[2], Margarita Trobos[2,5], Jaan Hong[3], Thomas J. Webster[4], Peter Thomsen[2] and Martin Andersson[1]

Corresponding author Email: martin.andersson@chalmers.se

a) has contributed equally to this work.

In order to ensure that the AMPs are covalently attached to the hydrogels and do not leak out during long-term washing, fluorescent-tagged AMPs (5(6) carboxyfluorescein-AMP) was covalently bonded and physically adsorbed onto hydrogels and washed for 3 weeks (Figure S1). It was shown that AMPs that were covalently bonded onto hydrogels did not leak out upon washing with milli-Q water for up to three weeks while the physically absorbed AMPs lost the whole AMP content by the end of the experiment.

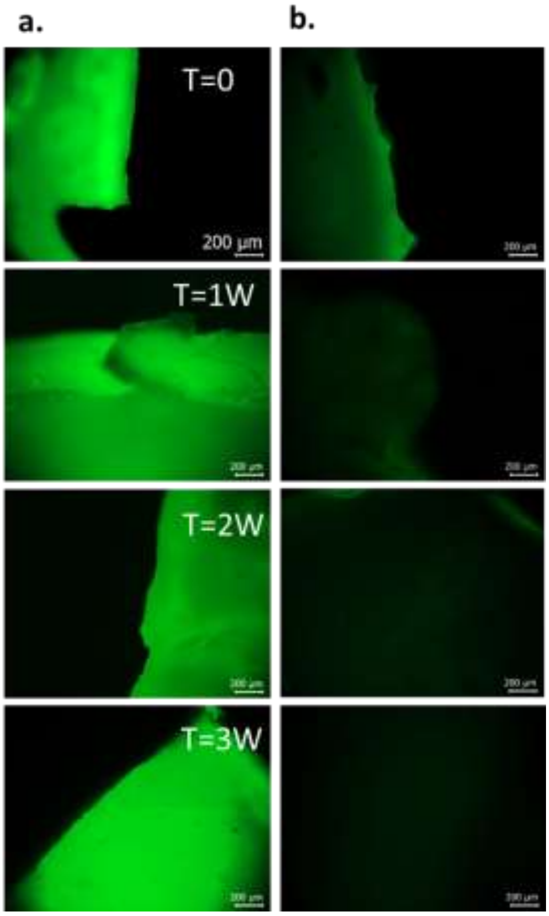

Figure S1. 5(6) carboxyfluorescein-AMP (green fluorescence): (a)covalently bonded using EDC-NHS coupling and (b) physically absorbed onto hydrogels (by submerging the hydrogels into AMP solution), washed in milli-Q water up to 3 weeks. The mili-Q water was changed twice per day and the surfaces were put back into the mili-Q water after imaging with a fluorescent microscope. The microscope used was a Zeiss Axio Imager Z2m.
